# Diagnostic Accuracy of Point-of-Care Lung Ultrasonography and Chest Radiography in Adults With Symptoms Suggestive of Acute Decompensated Heart Failure: A Systematic Review and Meta-analysis

**DOI:** 10.1001/jamanetworkopen.2019.0703

**Published:** 2019-03-15

**Authors:** Anna M. Maw, Ahmed Hassanin, P. Michael Ho, Matthew D. F. McInnes, Angela Moss, Elizabeth Juarez-Colunga, Nilam J. Soni, Marcelo H. Miglioranza, Elke Platz, Kristen DeSanto, Anthony P. Sertich, Gerald Salame, Stacie L. Daugherty

**Affiliations:** 1 Division of Hospital Medicine, University of Colorado, Aurora; 2 Division of Cardiology, University of Colorado School of Medicine, Aurora; 3 Division of Cardiology, VA Eastern Colorado Health Care System, Aurora; 4 Department of Radiology, University of Ottawa, Ottawa, Ontario, Canada; 5 Clinical Epidemiology Program, Ottawa Hospital Research Institute, Ottawa, Ontario, Canada; 6 Adult and Child Consortium for Health Outcomes Research and Delivery Science, Aurora, Colorado; 7 Rocky Mountain Prevention Research Center, Aurora, Colorado; 8 Division of Pulmonary and Critical Care Medicine and Division of General and Hospital Medicine, The University of Texas School of Medicine at San Antonio, San Antonio; 9 Section of Hospital Medicine, South Texas Veterans Health Care System, San Antonio; 10 Institute of Cardiology/University Foundation of Cardiology, Porto Alegre, Brazil; 11 Department of Emergency Medicine, Brigham and Women's Hospital, Boston, Massachusetts; 12 Harvard Medical School, Boston, Massachusetts; 13 Health Sciences Library, University of Colorado, Aurora; 14 Division of Hospital Medicine, University of Texas Southwestern Medical Center, Dallas

## Abstract

**Importance:**

Standard tools used to diagnose pulmonary edema in acute decompensated heart failure (ADHF), including chest radiography (CXR), lack adequate sensitivity, which may delay appropriate diagnosis and treatment. Point-of-care lung ultrasonography (LUS) may be more accurate than CXR, but no meta-analysis of studies directly comparing the 2 tools was previously available.

**Objective:**

To compare the accuracy of LUS with the accuracy of CXR in the diagnosis of cardiogenic pulmonary edema in adult patients presenting with dyspnea.

**Data Sources:**

A comprehensive search of MEDLINE, Embase, and Cochrane Library databases and the gray literature was performed in May 2018. No language or year limits were applied.

**Study Selection:**

Study inclusion criteria were a prospective adult cohort of patients presenting to any clinical setting with dyspnea who underwent both LUS and CXR on initial assessment with imaging results compared with a reference standard ADHF diagnosis by a clinical expert after either a medical record review or a combination of echocardiography findings and brain-type natriuretic peptide criteria. Two reviewers independently assessed the studies for inclusion criteria, and disagreements were resolved with discussion.

**Data Extraction and Synthesis:**

Reporting adhered to the Cochrane Handbook for Systematic Reviews of Diagnostic Test Accuracy and the Preferred Reporting Items for Systematic Reviews and Meta-analyses guidelines. Two authors independently extracted data and assessed the risk of bias using a customized QUADAS-2 tool. The pooled sensitivity and specificity of LUS and CXR were determined using a hierarchical summary receiver operating characteristic approach.

**Main Outcomes and Measures:**

The comparative accuracy of LUS and CXR in diagnosing ADHF as measured by the differences between the 2 modalities in pooled sensitivity and specificity.

**Results:**

The literature search yielded 1377 nonduplicate titles that were screened, of which 43 articles (3.1%) underwent full-text review. Six studies met the inclusion criteria, representing a total of 1827 patients. Pooled estimates for LUS were 0.88 (95% Cl, 0.75-0.95) for sensitivity and 0.90 (95% Cl, 0.88-0.92) for specificity. Pooled estimates for CXR were 0.73 (95% CI, 0.70-0.76) for sensitivity and 0.90 (95% CI, 0.75-0.97) for specificity. The relative sensitivity ratio of LUS, compared with CXR, was 1.2 (95% CI, 1.08-1.34; *P* < .001), but no difference was found in specificity between tests (relative specificity ratio, 1.0; 95% CI, 0.90-1.11; *P* = .96).

**Conclusions and Relevance:**

The findings suggest that LUS is more sensitive than CXR in detecting pulmonary edema in ADHF; LUS should be considered as an adjunct imaging modality in the evaluation of patients with dyspnea at risk of ADHF.

## Key Points

**Question:**

How does the accuracy of lung ultrasound compare with chest radiography for diagnosing cardiogenic pulmonary edema in patients presenting to any clinical setting with dyspnea?

**Findings:**

In this systematic review with meta-analysis of 6 prospective cohort studies representing 1827 patients, lung ultrasonography was found to be more sensitive than chest radiography for the detection of cardiogenic pulmonary edema and had comparable specificity.

**Meaning:**

Lung ultrasonography appeared to be useful as an adjunct imaging study in patients presenting with dyspnea at risk for heart failure.

## Teaser

This systematic review and meta-analysis compares the accuracy of lung ultrasonography vs chest radiography in the diagnosis of pulmonary edema in acute decompensated heart failure in adults.

## Introduction

Acute decompensated heart failure (ADHF) is the primary cause in up to 40% of older adults presenting with dyspnea,^1^ one of the leading reasons for emergency department visits in the United States.^2^ The diagnostic workup for ADHF can be challenging and often requires several tests. The insensitivity of guideline-recommended tools for diagnosing ADHF, such as chest radiography (CXR), physical examination, and brain-type natriuretic peptide (BNP),^3,4^ is known to delay treatment, which is associated with an increase in mortality.^5,6^ In particular, the sensitivity of CXR in detecting pulmonary edema is limited, with 20% being a false-negative.^7,8^ Given the limitations of current tools to diagnose ADHF, the National Heart, Lung, and Blood Institute Working Group on Emergency Department Management of Acute Heart Failure has prioritized the development of new techniques for the diagnosis and monitoring of ADHF.^9^

Point-of-care lung ultrasonography (LUS), which is performed and interpreted at the bedside by the treating clinician, has emerged as a practical diagnostic tool for several lung pathologies. Growing evidence indicates that LUS has comparable or superior accuracy over CXR for many of the most common causes of dyspnea.^10,11,12^ Sonographic B-lines are hyperechoic reverberation artifacts that extend vertically from the pleural surface to the bottom of the screen and move synchronously with lung sliding.^13^ The number of B-lines seen on LUS has been shown to offer a semiquantitative measure of extravascular lung water content.^14,15,16^ However, data on the diagnostic accuracy of LUS for cardiogenic pulmonary edema are conflicting, with reported sensitivity ranging from 57%^17^ to higher than 95%.^18,19^ Given its potential advantages over CXR, including its ease of acquisition, immediate availability of results, and evidence of comparable accuracy, LUS could have important implications for standard of care in the evaluation of patients with dyspnea at risk for ADHF.

Although previous systematic reviews have focused on the accuracy of various tools to diagnose ADHF in patients presenting with dyspnea,^20,21^ none of them has directly compared the accuracy of LUS with CXR accuracy. The objective of this study was to perform a systematic review with meta-analysis to determine the comparative accuracy of LUS and CXR for the diagnosis of cardiogenic pulmonary edema in patients presenting with dyspnea.

## Methods

This systematic review was conducted in compliance with the recommendations from the *Cochrane Handbook for Systematic Reviews of Diagnostic Test Accuracy*.^22^ This study followed the Preferred Reporting Items for Systematic Reviews and Meta-analyses (PRISMA) reporting guideline.^23^ The study protocol was registered on PROSPERO^24^ prior to study selection.

### Search Strategy

A comprehensive literature search of MEDLINE, Embase, and Cochrane Library databases was performed in November 2017 and was updated in May 2018. A search of the gray literature was also performed through May 2018 and included conference proceedings from 2014 to 2018 of the American College of Chest Physicians (Annual Meeting Abstract supplements), American Heart Association (Scientific Sessions abstracts), and American College of Cardiology (Annual Scientific Sessions abstracts) as well as from ClinicalTrials.gov and ProQuest Dissertations and Theses databases.

The search strategy (eTable 1 in the Supplement) was developed by our principal investigator (A.M.M.) in collaboration with a medical librarian (K.D.). No language or year limits were applied. Retrieved titles and abstracts were independently reviewed by 2 of us (G.S. and A.S.). Full-text versions of relevant studies were retrieved for further evaluation, and the inclusion criteria were applied independently by 2 of us (A.M.M. and A.H.). The inclusion criteria required studies to be prospective cohorts of adult patients presenting with acute dyspnea to any clinical setting in which both LUS and CXR were performed on initial assessment of all patients. In addition, a reference standard of an ADHF diagnosis had to be made by an independent expert after a medical record audit^20^ or a combination of echocardiographic findings and BNP criteria. Sufficient data to calculate both sensitivity and specificity were also required. Study participants could not be a subset of patients from another included paper (ie, no overlapping samples or duplicate patients).

### Data Extraction and Quality Assessment

Two of us (A. M. and A. H.) independently extracted data by using standardized data-extraction sheets. When pertinent data were not included in a manuscript, the study’s corresponding author was contacted. If no reply was received or the authors did not have the requested information, the data were labeled as *not specified*.

Data were extracted into 3 tables. Table 1 and Table 2 show relevant study characteristics. The numeric data necessary to create 2-by-2 contingency tables were extracted into a third table, from which sensitivity and specificity were then calculated. In the case of multiple individuals interpreting the LUS within the primary study, we determined the mean LUS results (ie, the number of B-lines) of all interpreters. In the case of an individual study reporting more than 1 threshold, our a priori plan was to use the threshold closest to those of all other included studies.

**Table 1.** Study Characteristics

Source	Geographic Location	Enrollment Period	Study-Specific Exclusion Criteria	No. of Participants		Patients With ADHF, No. (%)	Age, Mean (SD) or Median (IQR), y	% of Female Participants
				Enrolled	Analyzed			
**Setting: Emergency Department**									
Baker et al,^25^ 2013	Australia	March 2011 to February 2012	Patients needing active resuscitation; symptoms associated with trauma	230	204	41 (20)	Median (IQR): 76 (15)	46
Öhman et al,^18^ 2017	The Netherlands	July 2014 to January 2015	Age <18 y; history of pulmonary fibrosis; mitral stenosis or a prosthetic mitral position on echo	100	100	52 (52)	Mean (SD): 71 (15)	Not specified
Pivetta et al,^26^ 2015	Italy	October 2010 to September 2012	Traumatic injury; patients invasively ventilated at the time of evaluation	1008	1005	463 (46)	Median (IQR): 77 (13)	46
Sartini et al,^17^ 2017	Italy	January 2011 and February 2013	Age <18 y; symptoms associated with trauma	255	236	114 (48)	Mean (SD): 80 (12)	54
**Setting: Internal Medicine Inpatient Ward**									
Perrone et al,^27^ 2017	Italy	December 2014 to June 2016	History of pulmonary cancer; history of fibrothorax or congenital lung diseases	150	130	80 (62)	Mean (SD): 81 (9)	54
Vitturi et al,^19^ 2011	Italy	November 2007 to March 2008	Lung cancer; fibrothorax; congenital pulmonary diseases; major thoracic surgery	152	152	68 (45)	Not specified	Not specified

Abbreviations: ADHF, acute decompensated heart failure; IQR, interquartile range.

**Table 2.** Index Test and Reference Characteristics

Source	Ultrasonography Machine and Transducer	No. and Qualifications of Sonographer	Length of LUS Clip, s	No. of Lung Zones Scanned per Hemi-Thorax	Interrater Reliability κ (95% CI)	Threshold for a Positive LUS	LUS Interpreter^a^	CXR Technique	CXR Interpreter	LUS/CXR Time Interval, h	Description of Reference Diagnosis Method
**Setting: Emergency Department**												
Baker et al,^25^ 2013	GE Logic-e portable ultrasonography (GE), with a 2-5 MHz curvilinear transducer	1 experienced, 11 novices	3	4	0.82 (0.72-0.92)^b^ and 0.70 (0.45-0.95)^c^	Positive if ≥3 B-lines were demonstrated in ≥2 areas bilaterally	Blinded LUS expert (read offline)	Postero-anterior for most patients	Radiologist (blinding incomplete)	<1, or within 2, providing that no active fluid management occurred in the intervening period	Medical record audit by a specialist cardiologist blinded to LUS results
Öhman et al,^18^ 2017	Philips CX 50, device (Philips), with phased array transducer	1 experienced sonographer, with >1000 examinations	5-10	2	1 (1.0,1.0)	Positive if ≥3 B-lines were demonstrated in ≥1 area bilaterally	Blinded sonographer (read at bedside)	Postero-anterior, sometimes with a lateral view	Radiologist (blinding unclear)	Immediately, not specified otherwise	If the following 2 criteria were fulfilled: “presence of heart disease on conventional echo and either a BNP of more than 400 ng/l or a BNP of more than 100 ng/l in combination with congestion on chest radiography”
Pivetta et al,^26^ 2015	Curvilinear transducer (5-3 MHz)	Multiple emergency department physicians; qualifications are not clear	5	3	0.94 (0.89-0.98)	Positive if ≥2 B-lines were demonstrated in ≥2 areas bilaterally	Blinded LUS expert (read offline)	Typically postero-anterior	Radiologist (blinding unclear)	1.5	Medical record audit by an emergency physician and a cardiologist blinded to LUS results
Sartini et al,^17^ 2017	Esaote MyLab30TM and MyLab70TM (Esaote), with convex array transducer (3.5–5 MHz)	Multiple emergency physicians with at least 50 previous supervised examinations	NA (still image)	6	Not reported	Positive if ≥2 B-lines were demonstrated in ≥2 areas bilaterally	Blinded sonographer (read at bedside)	Postero-anterior	Radiologist (blinding unclear)	2	Medical record audit, on discharge, by 2 cardiologist and 1 emergency physician
**Setting: Internal Medicine Inpatient Ward**												
Perrone et al,^27^ 2017	Esaote MyLab 5 sonograph (Esaote), with convex 3.5 MHz transducer	1 skilled operator	5	4	Not reported	Positive if ≥2 B-lines were demonstrated in ≥2 areas bilaterally	Blinded sonographer (read at bedside)	Postero-anterior	Radiologist (blinding unclear)	Median (IQR): 12 (5 - 18)	Medical record audit by an independent experienced reviewer
Vitturiet al,^19^ 2011	Toshiba Aplio XV (Toshiba), with 3.5 MHz convex transducer	2 internists trained and experienced in United States	10	3	Not reported	Positive when the number of B- lines was greater than 8	Blinded sonographer (read at bedside)	Postero-anterior	Radiologist (blinding unclear)	2-6	Medical record audit by medical experts in accordance with AHA guidelines

Abbreviations: AHA, American Heart Association; BNP, brain-type natriuretic peptide; CXR, chest radiography; IQR, interquartile range; LUS, lung ultrasonography; NA, not applicable.

^a^ All LUSs were interpreted either offline by an LUS expert blinded to all clinical data or bedside by a sonographer blinded to all clinical information except that which could not be blinded (ie, the physical appearance of the patient).

^b^ Estimate for interrater reliability between LUS experts.

^c^ Estimate for interrater reliability between LUS experts and novices.

### Assessment of Risk of Bias and Applicability

A customized QUADAS-2 tool^28^ was applied to assess the risk of bias and applicability to the research question. Two of us (A.M.M. and A.H.) applied the tool independently to all studies. Disagreements between us were resolved by discussion. Interrater agreement of QUADAS-2 ratings were assessed using Cohen κ statistic and Gwet AC1.^29,30^

Based on the QUADAS-2 tool, each article was evaluated for risk of bias in 4 domains. High risk of bias in each domain was defined as follows: (1) patient selection, the study enrollment was not consecutive or the study excluded patients that could introduce spectrum bias, which is the phenomenon that the performance of a diagnostic test will vary with the spectrum of severity of disease present in the cohort; (2) index test, the LUS or CXR results were interpreted without blinding to the reference standard or the other index test; (3) reference standard, the reference standard for ADHF diagnosis was interpreted without blinding to the results of the index tests (LUS or CXR); and (4) flow and timing, the interval between LUS and CXR exceeded 2 hours. eFigure 1 in the Supplement provides the QUADAS-2 tool used in this systematic review.

With regard to assessment of applicability, each article was evaluated for low and high concern for applicability to the research question. Using the patient selection, index test, and reference standard domains, we defined low applicability concern as follows: (1) patient selection, the patient presented to the health care setting with acute dyspnea; (2) index test, the CXR was performed according to the hospital’s standard procedure; (3) index test, the LUS was acquired according to the international evidence-based recommendations for point-of-care LUS (Volpicelli criteria: 2 lung fields with 3 or more B-lines present bilaterally^31^) or a modification of it; and (4) reference standard, the diagnosis was based on medical record audit by 1 or more attending physicians or by structural heart disease on echocardiography and on BNP greater than 100 ng/l (to convert to picogram/milliliter, multiply by 1.0) or N-terminal pro-BNP greater than 900 pg/mL.

### Data Analysis

The a priori intention was to attempt a meta-analysis using the hierarchical summary receiver operating characteristic (HSROC) curve model.^32,33^ The HSROC model is a statistically rigorous approach for the meta-analysis of diagnostic test accuracy studies.^34^ It assumes that an underlying receiver operating characteristic curve exists within each study.^35^ Pooled sensitivity and specificity were calculated from the parameter estimates of the HSROC model, as were positive and negative likelihood ratios. Positive and negative predictive values were calculated from pooled estimates of sensitivity and specificity as well as from pooled prevalence of the included studies. Using test type (LUS vs CXR) as a covariate, we performed an overall likelihood ratio test to evaluate the overall differences in sensitivity and specificity between index tests. Individual tests were also performed for differences in sensitivity and specificity between index tests. Statistical tests were performed as 2-sided tests with a *P =* .05 level of significance. The χ^2^ and Wald tests were used to determine *P* values. Meta-analysis was performed using the SAS metadas macro, version 9.4 (SAS Institute Inc).

Individual study results for sensitivity and specificity were plotted on a forest plot to visually assess and explore study variability. With regard to the explanation for the variability seen between studies, we identified a priori 2 possible sources of variability: (1) spectrum of disease, which is the range of ADHF disease severity as measured by the need for positive pressure ventilation, intensive care unit admission, or outpatient care, and (2) threshold effect, which is the criteria used to define a positive test result. We assessed for threshold effect using a visual inspection of summary receiver operating characteristic (SROC) curves and Spearman rank correlation for sensitivity and 1-specificity.^36^ Distribution of the study results closely along the estimated SROC curve suggests that differences in the threshold for a positive result among studies explain some of the variability observed. Publication bias was not assessed as no accepted method exists for its evaluation in a meta-analysis of diagnostic test accuracy studies.^32^

## Results

### Search

**Figure 1.**
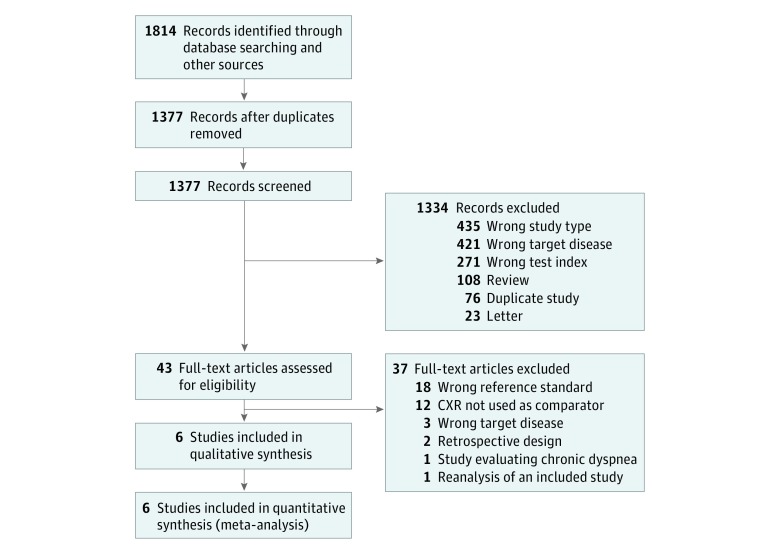
Flow Diagram Outlining Search Through Inclusion Process CXR indicates chest radiography.

Figure 1 is a study flow diagram detailing search results and study inclusion. The search identified 1377 nonduplicate titles that underwent screening, of which 43 articles (3.1%) underwent full-text review. After application of the exclusion criteria, we identified 6 studies eligible for data extraction, representing a total of 1827 patients.^17,18,19,25,26,27^ Figure 1 provides reasons for exclusion and the number of studies excluded under each reason.

### Study Characteristics

Table 1 summarizes the basic characteristics of the included studies. Four studies (67%) were conducted in emergency department cohorts and 2 studies (33%) in internal medicine ward patients who initially presented to the emergency department with dyspnea. Table 2 summarizes relevant index test and reference standard characteristics for included studies.

The number of LUS operators per study ranged from 1 to at least 12 (some studies reported multiple sonographers but did not specify the exact number), with each patient assessed by a single sonographer in 5 (83%) of 6 studies. In Vitturi et al,^19^ the exception study, LUS was performed twice by 2 different sonographers on each patient to assess interoperator variability. Three studies (50%) reported the LUS interrater agreement as κ, which ranged from 0.70 to 1.00. In 4 studies (67%), the sonographers acquiring the images were blinded to all clinical information except that which was seen at the bedside (ie, physical appearance of the patient) and interpreted the ultrasonography images. In 2 studies (33%), an LUS expert blinded to all clinical information interpreted previously recorded images. The length of the LUS image clip ranged from a still image to 10 seconds, and the number of lung zones examined per patient ranged from 4 to 12. All studies used the Volpicelli criteria^31^ as the threshold for positive LUS or a modification of it.

Chest radiographs were typically obtained using postero-anterior view and interpreted by a radiologist in all studies. Blinding of the radiologist to clinical information was unclear in all included studies. The interval time between LUS and CXR ranged from fewer than 1 to 12 hours.

**Figure 2.**
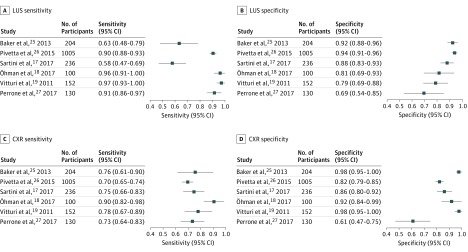
Forest Plots for Lung Ultrasonography and Chest Radiography CXR indicates chest radiography; LUS, lung ultrasonography.

Five (83%) of 6 studies used expert medical record review as the reference standard. The remaining study, Öhman et al,^18^ used echocardiography, BNP, and CXR criteria as the reference standard. Figure 2 summarizes the estimate of sensitivity and specificity obtained from each study.^17,18,19,25,26,27^ Vitturi et al^19^ did not offer a global estimate of CXR sensitivity and specificity but instead described test characteristics of particular CXR findings. In this case, we used the highest estimates for sensitivity and specificity of CXR that were reported in the study. The study authors reported no conflicts of interests.

### Primary Outcome

For LUS, estimates of sensitivity ranged from 58% to 97% and specificity ranged from 69% to 94% among included studies. For CXR, estimates ranged from 70% to 90% for sensitivity and 61% to 98% for specificity (Figure 2).

Pooled estimates for LUS, calculated from the parameter estimates of the HSROC model, were 0.88 (95% Cl, 0.75-0.95) for sensitivity and 0.90 (95% Cl, 0.88-0.92) for specificity. In contrast, for CXR the pooled estimate for sensitivity was 0.73 (95% Cl, 0.70-0.76) and for specificity was 0.90 (95% Cl, 0.75-0.97).

The overall and the individual tests performed on the HSROC model found the relative sensitivity ratio of LUS, compared with CXR, to be 1.2 (95% CI, 1.08-1.34; *P* < .001) but found no difference in specificity between tests (relative specificity ratio, 1.0; 95% CI, 0.90-1.11; *P* = .96). Table 3 shows additional test characteristics for CXR and LUS calculated from the HSROC model and from pooled ADHF prevalence in all included studies.

**Table 3.** Test Characteristics of Lung Ultrasonography and Chest Radiography^a^

Index Test	Positive Likelihood Ratio (95% CI)	Negative Likelihood Ratio (95% CI)	PPV (95% CI)^b^	NPV (95% CI)^b^	Sensitivity (95% CI)^c^	Specificity (95% CI)^c^
CXR	7.36 (2.70-20.07)	0.30 (0.26-0.35)	0.86 (0.69-0.95)	0.80 (0.75-0.83)	0.73 (0.70-0.76)	0.90 (0.75-0.97)
LUS	8.63 (6.93-10.74)	0.14 (0.06-0.29)	0.88 (0.83-0.90)	0.90 (0.81-0.95)	0.88 (0.75-0.95)	0.90 (0.88-0.92)

Abbreviations: LUS, lung ultrasonography; CXR, chest radiography; NPV, negative predictive value; PPV, positive predictive value.

^a^ All estimates calculated using the hierarchical summary receiver operating characteristic model.

^b^ Taking into account the minimum (20%) and maximum (62%) prevalence across studies for LUS, the PPV ranged from 0.68 to 0.93 and the NPV ranged from 0.82 to 0.97. For CXR, the PPV ranged from 0.65 to 0.92 and the NPV ranged from 0.68 to 0.93.

^c^ Sensitivity analysis was performed using the highest sensitivity and corresponding specificity and again for the highest specificity and corresponding sensitivity for CXR parameters in Vitturi et al.^19^ The results did not differ from the main analysis in that relative sensitivity and the log ratio test remained statistically significant.

### Assessment of Risk of Bias and Applicability

The results of the risk of bias and applicability concern assessment of individual studies using the QUADAS-2 tool are shown in eTable 2 in the Supplement. Cohen κ was 0.36 (95% CI, 0.08-0.64) and Gwet AC1 was 0.61 (95% CI, 0.40-0.83), indicating fair to substantial interrater agreement beyond that of chance of QUADAS-2 assessments.^29^ These results are within the range of findings in other studies that used QUADAS-2 as the quality assessment tool.^28^ With regard to the patient selection domain, enrollment in 3 of the 6 trials (50%) was not consecutive but rather a convenience sample based on the availability of an LUS sonographer. Although it is possible that convenience sampling introduced an element of spectrum bias (ie, sicker patients may present at night rather than the day), it is unlikely to have greatly affected the relative performance of LUS to CXR. Given that all CXRs were interpreted by a radiologist, available expertise in CXR interpretation was unlikely to have contributed to a relative difference in accuracy between imaging modalities for the included studies with convenience samples.

The reference standard domain was found to be at high risk of bias across all studies. Study adjudicators for 5 of the 6 included studies (83%) had access to CXR results during medical record audit, suggesting review bias. The 1 remaining study, Öhman et al,^18^ had CXR incorporated into the reference standard criteria, demonstrating incorporation bias. Thus, reference standard results were at high risk of bias, likely leading to overestimates in CXR accuracy across all studies.

Both Perrone et al^27^ and Vitturi et al^19^ were found to be at high risk of bias in the flow and timing domain, with CXR performed several hours before LUS. Given that symptomatic patients may have received diuretics in the interval between index tests, we expected this intervention to underestimate LUS sensitivity compared with CXR.

With regard to the applicability assessment, only 1 study met the criteria for high concern. Sartini et al^17^ included patients who did not match the review question for the patient selection domain, as 50 study participants (37%) were treated for ADHF prior to undergoing CXR and LUS. Therefore, the imaging study was not used in the initial decision to diagnose or treat ADHF. No other concerns were found regarding applicability to the research question in any other domain.

Although not part of the QUADAS- 2 assessment, unclear or incomplete blinding of LUS and CXR interpreters to clinical data occurred. In 4 of the 6 included studies (67%), LUS was interpreted by the sonographer who obtained the images; although the sonographers were blinded to all other clinical information, they may have seen information at the bedside that could have affected their interpretation. Similarly, to what extent the radiologists were blinded to clinical data was unclear in all but 2 studies: Baker et al^25^ reported that radiologists routinely reviewed previous chest imaging, and Öhman et al^18^ reported blinding of radiologists to laboratory data and final diagnosis only.

### Assessment of Variability

Visual inspection of the forest plot for LUS sensitivity revealed 2 potential outliers, Baker et al^25^ and Sartini et al,^17^ both of which reported LUS sensitivities lower than almost all of the other studies on this topic. Visual inspection of the SROC curves (eFigure 2 in the Supplement) demonstrated that the distribution of accuracy estimates found in each study followed the best-fit SROC curve, supporting the idea that differences in the threshold used to define a positive result between studies explain some of the variability observed between LUS results. Spearman rank correlation coefficient (ρ = 0.6) provided further evidence of the threshold effect contributing to the variability seen between studies for LUS but not for CXR. We were unable to evaluate variability based on spectrum of disease as no studies of outpatient or intensive care unit cohorts met the inclusion criteria.

A possible outlier for CXR sensitivity was the high estimate in Öhman et al,^18^ which may be associated with incorporation bias: CXR was part of the reference standard criteria in that study.

A possible outlier for CXR specificity was the low estimate in Perrone et al,^27^ which may be explained by 10% of patients having both pulmonary and cardiac processes associated with their abnormal imaging findings. Consistent with this premise, the LUS specificity estimate was lower in Perrone et al,^27^ compared with other included studies.

## Discussion

Among adults presenting to a hospital setting for acute dyspnea, a 15% absolute difference in sensitivity was found between LUS and CXR (0.88 vs 0.73), favoring LUS, but no statistically significant difference in specificity was found for the detection of pulmonary edema from ADHF. Specifically, for every 100 patients presenting with dyspnea owing to cardiogenic pulmonary edema, LUS can diagnose 15 more cases than CXR without an increase in the number of false-positives. In addition, we identified threshold effect to be a likely contributor to the variability seen in LUS accuracy results. The estimates for sensitivity and specificity are in agreement with other systematic reviews that evaluated the accuracy of LUS in the diagnosis of cardiogenic pulmonary edema.^37,38,39^ To our knowledge, this is the first study that compared LUS accuracy with that of CXR by including only studies that performed both tests in all study participants, thereby minimizing the risk of bias and confounding owing to the differences in reference standards and study design. We chose this methodologic approach to prioritize internal validity over statistical power, knowing that it would result in fewer included studies. In spite of this tradeoff, the analysis of these data demonstrates that LUS, compared with CXR, has better sensitivity in the detection of pulmonary edema from ADHF and has comparable specificity.

The high variability present between LUS sensitivity results appears to be driven by the lower estimates of LUS sensitivity found in Sartini et al^17^ and Baker et al.^25^ In these studies, LUS was acquired using a still image or a 3-second video clip. It has been shown that shorter clips can underestimate the number of B-lines seen.^40,41^ The short clip length used in both Sartini et al^17^ and Baker et al^25^ studies may explain their lower estimates of LUS sensitivity. In addition, in Sartini et al,^17^ 50 patients (37%) received diuretic therapy prior to presentation to the emergency department. In a subgroup analysis that excluded these patients, LUS sensitivity was found to be 83% and specificity was found to be 86%, whereas CXR was found to be 64% sensitive and 94% specific. Signs of pulmonary edema seen on CXR are known to lag in resolution.^42^ In contrast, LUS findings may be more responsive to dynamic changes in volume status.^43,44,45^ Therefore, the low sensitivity of LUS in the main analysis may have been associated with the resolution of LUS abnormalities from diuretics administered prior to LUS testing. The test characteristics in the post hoc subgroup analysis that excluded those with prehospital diuretics are more consistent with the available evidence on the accuracy of LUS^37,38,39^

In addition to improved accuracy, LUS may offer other benefits compared with CXR, including avoidance of ionizing radiation and immediate availability of results. Lung ultrasonography has also be shown to be easy for clinicians to learn, perform, and interpret.^37,46,47,48,49^ As presented in the clinical practice guidelines, the role of CXR in the evaluation of ADHF is, in part, to assess other causes of dyspnea. Because LUS has been shown to offer comparable or superior accuracy over many of the other most common causes of dyspnea,^10,11,12,50^ it has the potential to become an initial imaging modality in the evaluation of patients with dyspnea. Future prospective studies are needed to determine if the use of LUS in the initial evaluation of adults presenting with dyspnea improves diagnosis, treatment, and outcomes of patients with ADHF.

### Limitations

The main limitation of this systematic review with meta-analysis is the small number of included studies. Including only studies that evaluated the accuracy of both LUS and CXR to minimize bias contributed to the small number of included studies. This limited inclusion, in turn, decreased the precision of our estimates as well as our ability to formally evaluate for possible causes of variability, including clinical setting, using subgroup analysis, and meta-regression. However, on visual inspection of all forest plots (Figure 2), no clear evidence of a difference in test characteristic estimates was found between emergency department and inpatient cohorts for either imaging modality.

Another important limitation is the risk of bias introduced by the lack of blinding of outcome adjudicators to CXR in 5 of the studies and by the incorporation of CXR as part of the reference standard in the remaining study. Thus, the direction of bias across all included studies likely favors CXR. In addition, these results are generalizable only to patients presenting to the hospital with acute dyspnea. The study is representative of a large proportion of the studies investigating LUS accuracy for cardiogenic pulmonary edema.^37^

## Conclusions

The findings suggest that LUS is as specific and more sensitive than CXR in the identification of cardiogenic pulmonary edema. Given the potential advantages of its use, LUS should be considered as an adjunct imaging modality in the evaluation of patients with dyspnea at risk of ADHF.

## Notes

Supplement. **eTable 1.** Search Strategies **eTable 2.** QUADAS-2 Assessment of Methodologic Quality of Included Studies **eFigure 1.** QUADAS-2 Tool to Assess for Risk of Bias and Applicability **eFigure 2.** Summary Receiver Operating Characteristic Curves for Lung Ultrasound and Chest X-ray

## References

[1] Ray P, Birolleau S, Lefort Y, Acute respiratory failure in the elderly: etiology, emergency diagnosis and prognosis. Crit Care. 2006;10(3):. doi:10.1186/cc4926 16723034 PMC1550946

[2] Rui PKK National Hospital Ambulatory Medical Care Survey: 2014 emergency department summary tables. https://www.cdc.gov/nchs/data/nhamcs/web_tables/2014_ed_web_tables.pdf. Accessed June 14, 2018.

[3] Yancy CW, Jessup M, Bozkurt B, ; Writing Committee Members; American College of Cardiology Foundation/American Heart Association Task Force on Practice Guidelines 2013 ACCF/AHA guideline for the management of heart failure: a report of the American College of Cardiology Foundation/American Heart Association Task Force on practice guidelines. Circulation. 2013;128(16):e240-. doi:10.1161/CIR.0b013e31829e8776 23741058

[4] Ponikowski P, Voors AA, Anker SD, 2016 ESC Guidelines for the Diagnosis and Treatment of Acute and Chronic Heart Failure. Rev Esp Cardiol (Engl Ed). 2016;69(12):1167. doi:10.1016/j.rec.2016.11.005 27894487

[5] Matsue Y, Damman K, Voors AA, Time-to-furosemide treatment and mortality in patients hospitalized with acute heart failure. J Am Coll Cardiol. 2017;69(25):3042-3051. doi:10.1016/j.jacc.2017.04.042 28641794

[6] Peacock WF, Emerman C, Costanzo MR, Diercks DB, Lopatin M, Fonarow GC Early vasoactive drugs improve heart failure outcomes. Congest Heart Fail. 2009;15(6):256-264. doi:10.1111/j.1751-7133.2009.00112.x 19925503

[7] Collins SP, Lindsell CJ, Storrow AB, Abraham WT; ADHERE Scientific Advisory Committee, Investigators and Study Group Prevalence of negative chest radiography results in the emergency department patient with decompensated heart failure. Ann Emerg Med. 2006;47(1):13-18. doi:10.1016/j.annemergmed.2005.04.003 16387212

[8] Mueller-Lenke N, Rudez J, Staub D, Use of chest radiography in the emergency diagnosis of acute congestive heart failure. Heart. 2006;92(5):695-696. doi:10.1136/hrt.2005.074583 16159971 PMC1860911

[9] Peacock WF, Braunwald E, Abraham W, National Heart, Lung, and Blood Institute working group on emergency department management of acute heart failure: research challenges and opportunities. J Am Coll Cardiol. 2010;56(5):343-351. doi:10.1016/j.jacc.2010.03.051 20650354

[10] Yousefifard M, Baikpour M, Ghelichkhani P, Screening performance characteristic of ultrasonography and radiography in detection of pleural effusion; a meta-analysis. Emerg (Tehran). 2016;4(1):1-10. 26862542 PMC4744606

[11] Chavez MA, Shams N, Ellington LE, Lung ultrasound for the diagnosis of pneumonia in adults: a systematic review and meta-analysis. Respir Res. 2014;15:50. doi:10.1186/1465-9921-15-50 24758612 PMC4005846

[12] Alrajab S, Youssef AM, Akkus NI, Caldito G Pleural ultrasonography versus chest radiography for the diagnosis of pneumothorax: review of the literature and meta-analysis. Crit Care. 2013;17(5):R208. doi:10.1186/cc13016 24060427 PMC4057340

[13] Lichtenstein D, Mézière G, Biderman P, Gepner A, Barré O The comet-tail artifact: an ultrasound sign of alveolar-interstitial syndrome. Am J Respir Crit Care Med. 1997;156(5):1640-1646. doi:10.1164/ajrccm.156.5.96-07096 9372688

[14] Jambrik Z, Monti S, Coppola V, Usefulness of ultrasound lung comets as a nonradiologic sign of extravascular lung water. Am J Cardiol. 2004;93(10):1265-1270. doi:10.1016/j.amjcard.2004.02.012 15135701

[15] Agricola E, Bove T, Oppizzi M, “Ultrasound comet-tail images”: a marker of pulmonary edema: a comparative study with wedge pressure and extravascular lung water. Chest. 2005;127(5):1690-1695. doi:10.1378/chest.127.5.1690 15888847

[16] Baldi G, Gargani L, Abramo A, Lung water assessment by lung ultrasonography in intensive care: a pilot study. Intensive Care Med. 2013;39(1):74-84. doi:10.1007/s00134-012-2694-x 23052950

[17] Sartini S, Frizzi J, Borselli M, Which method is best for an early accurate diagnosis of acute heart failure? Comparison between lung ultrasound, chest X-ray and NT pro-BNP performance: a prospective study. Intern Emerg Med. 2017;12(6):861-869. doi:10.1007/s11739-016-1498-3 27401330

[18] Öhman J, Harjola VP, Karjalainen P, Lassus J Rapid cardiothoracic ultrasound protocol for diagnosis of acute heart failure in the emergency department [published online October 3, 2017]. Eur J Emerg Med. doi:10.1097/MEJ.0000000000000499 28984662

[19] Vitturi N, Soattin M, Allemand E, Simoni F, Realdi G Thoracic ultrasonography: a new method for the work-up of patients with dyspnea(). J Ultrasound. 2011;14(3):147-151. doi:10.1016/j.jus.2011.06.009 23396858 PMC3558237

[20] Martindale JL, Wakai A, Collins SP, Diagnosing acute heart failure in the emergency department: a systematic review and meta-analysis. Acad Emerg Med. 2016;23(3):223-242. doi:10.1111/acem.12878 26910112

[21] Wang CS, FitzGerald JM, Schulzer M, Mak E, Ayas NT Does this dyspneic patient in the emergency department have congestive heart failure? JAMA. 2005;294(15):1944-1956. doi:10.1001/jama.294.15.1944 16234501

[22] Deeks JJBP, Gatsonis C *Cochrane Handbook for Systematic Reviews of Diagnostic Test Accuracy, Version 1.0.0* London, UK: The Cochrane Collaboration; 2013 https://methods.cochrane.org/sdt/handbook-dta-reviews. Accessed June 12, 2018.

[23] McInnes MDF, Moher D, Thombs BD, ; the PRISMA-DTA Group Preferred Reporting Items for a Systematic Review and Meta-analysis of Diagnostic Test Accuracy Studies: The PRISMA-DTA Statement. JAMA. 2018;319(4):388-396. doi:10.1001/jama.2017.19163 29362800

[24] Maw A Accuracy of lung ultrasound versus chest x-ray for diagnosing pulmonary edema. PROSPERO. http://www.crd.york.ac.uk/PROSPERO/display_record.php?ID=CRD42017067355. Accessed June 12, 2018.

[25] Baker K, Mitchell G, Thompson AG, Stieler G Comparison of a basic lung scanning protocol against formally reported chest x-ray in the diagnosis of pulmonary oedema. Australas J Ultrasound Med. 2013;16(4):183-189. doi:10.1002/j.2205-0140.2013.tb00245.x 28191195 PMC5030057

[26] Pivetta E, Goffi A, Lupia E, ; SIMEU Group for Lung Ultrasound in the Emergency Department in Piedmont Lung ultrasound-implemented diagnosis of acute decompensated heart failure in the ED: a SIMEU multicenter study. Chest. 2015;148(1):202-210. doi:10.1378/chest.14-2608 25654562

[27] Perrone T, Maggi A, Sgarlata C, Lung ultrasound in internal medicine: a bedside help to increase accuracy in the diagnosis of dyspnea. Eur J Intern Med. 2017;46:61-65. doi:10.1016/j.ejim.2017.07.034 28793969

[28] Whiting PF, Rutjes AW, Westwood ME, ; QUADAS-2 Group QUADAS-2: a revised tool for the quality assessment of diagnostic accuracy studies. Ann Intern Med. 2011;155(8):529-536. doi:10.7326/0003-4819-155-8-201110180-00009 22007046

[29] Wongpakaran N, Wongpakaran T, Wedding D, Gwet KL A comparison of Cohen’s Kappa and Gwet’s AC1 when calculating inter-rater reliability coefficients: a study conducted with personality disorder samples. BMC Med Res Methodol. 2013;13:61. doi:10.1186/1471-2288-13-61 23627889 PMC3643869

[30] Gwet KL Computing inter-rater reliability and its variance in the presence of high agreement. Br J Math Stat Psychol. 2008;61(Pt 1):29-48. doi:10.1348/000711006X126600 18482474

[31] Volpicelli G, Elbarbary M, Blaivas M, ; International Liaison Committee on Lung Ultrasound (ILC-LUS) for International Consensus Conference on Lung Ultrasound (ICC-LUS) International evidence-based recommendations for point-of-care lung ultrasound. Intensive Care Med. 2012;38(4):577-591. doi:10.1007/s00134-012-2513-4 22392031

[32] McInnes MD, Bossuyt PM Pitfalls of systematic reviews and meta-analyses in imaging research. Radiology. 2015;277(1):13-21. doi:10.1148/radiol.2015142779 26402491

[33] Rutter CM, Gatsonis CA Regression methods for meta-analysis of diagnostic test data. Acad Radiol. 1995;2(suppl 1):S48-S56. 9419705

[34] Rutter CM, Gatsonis CA A hierarchical regression approach to meta-analysis of diagnostic test accuracy evaluations. Stat Med. 2001;20(19):2865-2884. doi:10.1002/sim.942 11568945

[35] Macaskill PGC, Deeks JJ, Harbord RM, Takwoingi Y Analysing and Presenting Results. Cochrane Handbook for Systematic Reviews of Diagnostic Test Accuracy Version 1.0. London, UK: The Cochrane Collaboration; 2010.

[36] Moses LE, Shapiro D, Littenberg B Combining independent studies of a diagnostic test into a summary ROC curve: data-analytic approaches and some additional considerations. Stat Med. 1993;12(14):1293-1316. doi:10.1002/sim.4780121403 8210827

[37] Al Deeb M, Barbic S, Featherstone R, Dankoff J, Barbic D Point-of-care ultrasonography for the diagnosis of acute cardiogenic pulmonary edema in patients presenting with acute dyspnea: a systematic review and meta-analysis. Acad Emerg Med. 2014;21(8):843-852. doi:10.1111/acem.12435 25176151

[38] McGivery K, Atkinson P, Lewis D, Emergency department ultrasound for the detection of B-lines in the early diagnosis of acute decompensated heart failure: a systematic review and meta-analysis. CJEM. 2018;20(3):343-352. doi:10.1017/cem.2018.27 29619917

[39] Wang Y, Shen Z, Lu X, Zhen Y, Li H Sensitivity and specificity of ultrasound for the diagnosis of acute pulmonary edema: a systematic review and meta-analysis. Med Ultrason. 2018;1(1):32-36. doi:10.11152/mu-1223 29400365

[40] Platz E, Pivetta E, Merz AA, Peck J, Rivero J, Cheng S Impact of device selection and clip duration on lung ultrasound assessment in patients with heart failure. Am J Emerg Med. 2015;33(11):1552-1556. doi:10.1016/j.ajem.2015.06.002 26123928 PMC4628583

[41] Pivetta E, Baldassa F, Masellis S, Bovaro F, Lupia E, Maule MM Sources of variability in the detection of B-lines, using lung ultrasound. Ultrasound Med Biol. 2018;44(6):1212-1216. doi:10.1016/j.ultrasmedbio.2018.02.018 29598962

[42] Assaad S, Kratzert WB, Shelley B, Friedman MB, Perrino A Jr Assessment of pulmonary edema: principles and practice. J Cardiothorac Vasc Anesth. 2018;32(2):901-914. doi:10.1053/j.jvca.2017.08.028 29174750

[43] Trezzi M, Torzillo D, Ceriani E, Lung ultrasonography for the assessment of rapid extravascular water variation: evidence from hemodialysis patients. Intern Emerg Med. 2013;8(5):409-415. doi:10.1007/s11739-011-0625-4 21590437

[44] Agricola E, Picano E, Oppizzi M, Assessment of stress-induced pulmonary interstitial edema by chest ultrasound during exercise echocardiography and its correlation with left ventricular function. J Am Soc Echocardiogr. 2006;19(4):457-463. doi:10.1016/j.echo.2005.11.013 16581487

[45] Noble VE, Murray AF, Capp R, Sylvia-Reardon MH, Steele DJR, Liteplo A Ultrasound assessment for extravascular lung water in patients undergoing hemodialysis: time course for resolution. Chest. 2009;135(6):1433-1439. doi:10.1378/chest.08-1811 19188552

[46] Mozzini C, Fratta Pasini AM, Garbin U, Cominacini L Lung ultrasound in internal medicine: training and clinical practice. Crit Ultrasound J. 2016;8(1):10. doi:10.1186/s13089-016-0048-6 27501700 PMC4977240

[47] Noble VE, Lamhaut L, Capp R, Evaluation of a thoracic ultrasound training module for the detection of pneumothorax and pulmonary edema by prehospital physician care providers. BMC Med Educ. 2009;9:3. doi:10.1186/1472-6920-9-3 19138397 PMC2631015

[48] Touw HR, Parlevliet KL, Beerepoot M, Lung ultrasound compared with chest X-ray in diagnosing postoperative pulmonary complications following cardiothoracic surgery: a prospective observational study. Anaesthesia. 2018;73(8):946-954. doi:10.1111/anae.14243 29529332 PMC6099367

[49] Gargani L, Frassi F, Soldati G, Tesorio P, Gheorghiade M, Picano E Ultrasound lung comets for the differential diagnosis of acute cardiogenic dyspnoea: a comparison with natriuretic peptides. Eur J Heart Fail. 2008;10(1):70-77. doi:10.1016/j.ejheart.2007.10.009 18077210

[50] Xia Y, Ying Y, Wang S, Li W, Shen H Effectiveness of lung ultrasonography for diagnosis of pneumonia in adults: a systematic review and meta-analysis. J Thorac Dis. 2016;8(10):2822-2831. doi:10.21037/jtd.2016.09.38 27867558 PMC5107554

