# Supplementary material for: Diagnostic Accuracy of Point-of-Care Lung Ultrasonography and Chest Radiography in Adults With Symptoms Suggestive of Acute Decompensated Heart Failure: A Systematic Review and Meta-analysis
Source: JAMA Netw Open. 2019 Mar 15;2(3):e190703. doi: 10.1001/jamanetworkopen.2019.0703 (PMC6484641; doi:10.1001/jamanetworkopen.2019.0703)
Supplement: Supplement. — eTable 1. Search Strategies eTable 2. QUADAS-2 Assessment of Methodologic Quality of Included Studies eFigure 1. QUADAS-2 Tool to Assess for Risk of Bias and Applicability eFigure 2. Summary Receiver Operating Characteristic Curves for Lung Ultrasound and Chest X-ray [file jamanetwopen-2-e190703-s001.pdf]

Supplementary Online Content

Maw AM, Hassanin A, Ho PM, et al. Diagnostic accuracy of point-of-care lung ultrasonography and chest radiography in adults with symptoms suggestive of acute decompensated heart failure: a systematic review and meta-analysis. *JAMA Netw Open.* 2019;2(3):e190703. doi:10.1001/jamanetworkopen.2019.0703

eTable 1. Search Strategies

eTable 2. QUADAS-2 Assessment of Methodologic Quality of Included Studies

eFigure 1. QUADAS-2 Tool to Assess for Risk of Bias and Applicability

eFigure 2. Summary Receiver Operating Characteristic Curves for Lung Ultrasound and Chest X-ray

This supplementary material has been provided by the authors to give readers additional information about their work.

©2019 Maw AM et al. *JAMA Network Open.*

eTable 1. Search Strategies

MEDLINE – search date 5/25/2018

#	**Search terms**	**Results**
1	exp Radiography, Thoracic/	36897
2	((chest or lung* or thora* or pulm*) adj4 (xray* or x-ray* or radiograph* or radiogram* or radiolog* or roentgen*)).mp.	80300
3	1 or 2	85152
4	exp Ultrasonography/	396639
5	((chest or lung* or thora* or pulm*) adj4 (sonogra* or ultrasound* or ultrasonic* or ultrasonogra* or ultra-sound* or ultra-sonic* or ultra-sonogra*)).mp.	4769
6	4 or 5	398844
7	exp Pulmonary Edema/	16605
8	((pulm* or lung* or cardiogenic) adj3 (edema* or oedema*)).mp.	26690
9	exp Extravascular Lung Water/	1111
10	((extravascular or extra-vascular) adj3 (pulm* or lung*) adj3 (water or fluid)).mp.	2188
11	wet lung.mp.	374
12	exp Heart Failure/	107511
13	((heart or cardiac) adj3 failure).mp.	189262
14	((preserved or reduced) adj3 ejection fraction).mp.	6738
15	interstitial syndrome.mp.	157
16	or/7-15	217154
17	3 and 6 and 16	783

Embase – search date 5/25/2018

#	**Search terms**	**Results**
1	'thorax radiography'/exp	164461
2	((chest or lung* or thora* or pulm*) NEAR/4 (xray* or x-ray* or radiograph* or radiogram* or radiolog* or roentgen*)):ab,ti	85116
3	#1 or #2	198228
4	'ultrasound'/exp	157205
5	((chest or lung* or thora* or pulm*) NEAR/4 (sonogra* or ultrasound* or ultrasonic* or ultrasonogra* or ultra-sound* or ultra-sonic* or ultra-sonogra*)):ab,ti	7491
6	#4 or #5	162337
7	'lung edema'/exp	39908
8	((pulm* or lung* or cardiogenic) NEAR/3 (edema* or oedema*)):ab,ti	27566
9	'lung extravascular fluid'/exp	1520
10	((extravascular or extra-vascular) NEAR/3 (pulm* or lung*) NEAR/3 (water or fluid)):ab,ti	2174
11	wet lung':ab,ti	458
12	'heart failure'/exp	440404
13	((heart or cardiac) NEAR/3 failure):ab,ti	248419
14	((preserved or reduced) NEAR/3 'ejection fraction'):ab,ti	13920
15	interstitial syndrome':ab,ti	226
16	#7 or #8 or #9 or #10 or #11 or #12 or #13 or #14 or #15	525423
17	#3 and #6 and #16	662

©2019 Maw AM et al. *JAMA Network Open.*

Cochrane – search date 5/25/2018

#	**Search terms**	**Results**
1	MeSH descriptor: [Radiography, Thoracic] explode all trees	414
2	(chest or lung* or thora* or pulm*) near/4 (xray* or x-ray* or radiograph* or radiogram* or radiolog* or roentgen*):ti,ab,kw (Word variations have been searched)	3008
3	#1 or #2	3027
4	MeSH descriptor: [Ultrasonography] explode all trees	13757
5	(chest or lung* or thora* or pulm*) near/4 (sonogra* or ultrasound* or ultrasonic* or ultrasonogra* or ultra-sound* or ultra-sonic* or ultra-sonogra*):ti,ab,kw (Word variations have been searched)	489
6	#4 or #5	14125
7	MeSH descriptor: [Pulmonary Edema] explode all trees	220
8	(pulm* or lung* or cardiogenic) near/3 (edema* or oedema*):ti,ab,kw (Word variations have been searched)	1062
9	MeSH descriptor: [Extravascular Lung Water] explode all trees	57
10	(extravascular or extra-vascular) near/3 (pulm* or lung*) near/3 (water or fluid):ti,ab,kw (Word variations have been searched)	196
11	"wet lung":ti,ab,kw (Word variations have been searched)	5
12	MeSH descriptor: [Heart Failure] explode all trees	7251
13	(heart or cardiac) near/3 failure:ti,ab,kw (Word variations have been searched)	20181
14	(preserved or reduced) near/3 "ejection fraction":ti,ab,kw (Word variations have been searched)	1344
15	"interstitial syndrome":ti,ab,kw (Word variations have been searched)	8
16	#7 or #8 or #9 or #10 or #11 or #12 or #13 or #14 or #15	21381
17	#3 and #6 and #16	41

©2019 Maw AM et al. *JAMA Network Open.*

Web of Science – search date 5/25/2018

#	**Search terms**	**Results**
1	TS=((chest or lung* or thora* or pulm*) NEAR/4 (xray* or x-ray* or radiograph* or radiogram* or radiolog* or roentgen*))	35350
2	TS=((chest or lung* or thora* or pulm*) NEAR/4 (sonogra* or ultrasound* or ultrasonic* or ultrasonogra* or ultra-sound* or ultra-sonic* or ultra-sonogra*))	5057
3	TS=((pulm* or lung* or cardiogenic) NEAR/3 (edema* or oedema*))	17812
4	TS=((extravascular or extra-vascular) NEAR/3 (pulm* or lung*) NEAR/3 (water or fluid))	1552
5	TS=("wet lung")	187
6	TS=((heart or cardiac) NEAR/3 failure)	215213
7	TS=((preserved or reduced)NEAR/3 "ejection fraction")	9856
8	TS=("interstitial syndrome")	215
9	#3 or #4 or #5 or #6 or #7 or #8	233998

Additional grey literature

ClinicalTrials.gov (searched 5/18/2017) –

- "Lung Ultrasound for Acute Dyspnea in Emergency Department" https://clinicaltrials.gov/ct2/show/NCT01287429
- "Diagnostic Value of Portable Ultrasound for Dyspneic Patient Support in the Emergency Department (VSCAN-DYSP)" https://clinicaltrials.gov/ct2/show/NCT01818882
- "Comparison Between Lung Ultrasound and Chest Radiography for Acute Dyspnea" https://clinicaltrials.gov/ct2/show/NCT02105207
- "Evaluation of the Impact of the Use of Pulmonary and Diaphragmatic Ultrasound the Clinical Decision-making in Respiratory Physiotherapy in Patients in ICU (USADEPT)" https://clinicaltrials.gov/ct2/show/NCT02881814
- "The Role of Lung Ultrasound During Perioperative Evaluation" https://clinicaltrials.gov/ct2/show/NCT03111641

ProQuest Dissertations and Theses (searched 5/18/2017) – 137 results, but 0 relevant: (((chest OR lung* OR thora* OR pulm*) NEAR/4 (xray* OR x-ray* OR radiograph* OR radiogram* OR radiolog* OR roentgen*))) AND (((chest OR lung* OR thora* OR pulm*) NEAR/4 (sonogra* OR ultrasound* OR ultrasonic* OR ultrasonogra* OR ultra-sound* OR ultra-sonic* OR ultra-sonogra*))) AND (((pulm* OR lung* OR cardiogenic) NEAR/3 (edema* OR oedema*)) OR ((heart OR cardiac) NEAR/3 (failure)) OR ((extravascular or extra-vascular) NEAR/3 (pulm* or

©2019 Maw AM et al. *JAMA Network Open.*

lung*) NEAR/3 (water or fluid)) OR wet lung OR interstitial syndrome OR ((preserved or reduced) NEAR/3))

eTable 2. QUADAS-2 Assessment of Methodologic Quality of Included Studies

Risk of Bias						Applicability Concerns			
**Study**	**Patient selection**	**Index test (LUS)**	**Index test (CXR)**	**Reference standard**	**Flow and timing**	**Patient Selection**	**Index Test (LUS)**	**Index Test (CXR)**	**Criterion Standard**
**Baker at al.[35]**	**High**	**Low**	**Low**	**High**	**Low**	**Low**	**Low**	**Low**	**Low**
**Ohman et al.[18]**	**Low**	**Low**	**Low**	**High**	**Low**	**Low**	**Low**	**Low**	**Low**
**Perrone at al.[36]**	**Low**	**Low**	**Low**	**High**	**High**	**Low**	**Low**	**Low**	**Low**
**Pivetta et al.[34]**	**High**	**Low**	**Low**	**High**	**Low**	**Low**	**Low**	**Low**	**Low**
**Sartini et al.[17]**	**High**	**Low**	**Low**	**High**	**Low**	**High**	**Low**	**Low**	**Low**
**Vitturi et al.[19]**	**Low**	**Low**	**Low**	**High**	**High**	**Low**	**Low**	**Low**	**Low**

LUS = lung ultrasound
CXR = chest x-ray

©2019 Maw AM et al. *JAMA Network Open.*

eFigure 1. QUADAS-2 Tool to Assess for Risk of Bias and Applicability

<u>Domain 1: Patient selection</u>

Risk of bias

Was a consecutive or random sample of patients enrolled? Yes/No/Unclear

Was a case-control design avoided? Yes/No/Unclear

Did the study avoid inappropriate exclusions? Yes/No/Unclear

Could the selection of patients have introduced bias? RISK: LOW/HIGH/UNCLEAR

Concerns regarding applicability

Is there concern that the included patients do not match the review question? CONCERN: LOW/HIGH/UNCLEAR

<u>Domain 2: Index test</u>

Risk of bias

Were the index test results interpreted without knowledge of the results of the reference standard? Yes/No/Unclear

If a threshold was used, was it pre-specified? Yes/No/Unclear

Could the conduct or interpretation of the index test have introduced bias? RISK: LOW/HIGH/UNCLEAR

Concerns regarding applicability

Is there concern that the index test, its conduct, or interpretation differ from the review question? CONCERN: LOW/HIGH/UNCLEAR

<u>Domain 3: Reference standard</u>

Risk of bias

Is the reference standard likely to correctly classify the target condition? Yes/No/Unclear

©2019 Maw AM et al. *JAMA Network Open.*

Were the reference standard results interpreted without knowledge of the results of the index test? Yes/No/Unclear

Could the reference standard, its conduct or its interpretation have introduced bias? RISK: LOW/HIGH/UNCLEAR

Concerns regarding applicability

Is there concern that the target condition as defined by the reference standard does not match the review question? CONCERN: LOW/HIGH/UNCLEAR

Domain 4: Flow and timing

Risk of bias

Was there an appropriate interval between index test(s) and reference standard? Yes/No/Unclear

Did all patients receive a reference standard? Yes/No/Unclear

Did patients receive the same reference standard? Yes/No/Unclear

Were all patients included in the analysis? Yes/No/Unclear

Could the patient flow have introduced bias? RISK: LOW/HIGH/UNCLEAR

©2019 Maw AM et al. *JAMA Network Open.*

eFigure 2. Summary Receiver Operating Characteristic Curves for Lung Ultrasound and Chest X-ray

A.

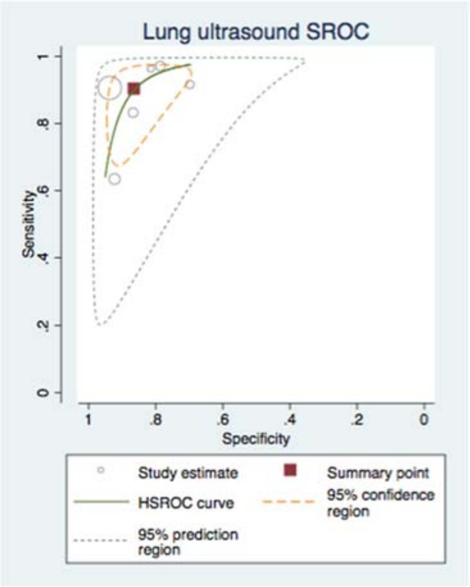

B.

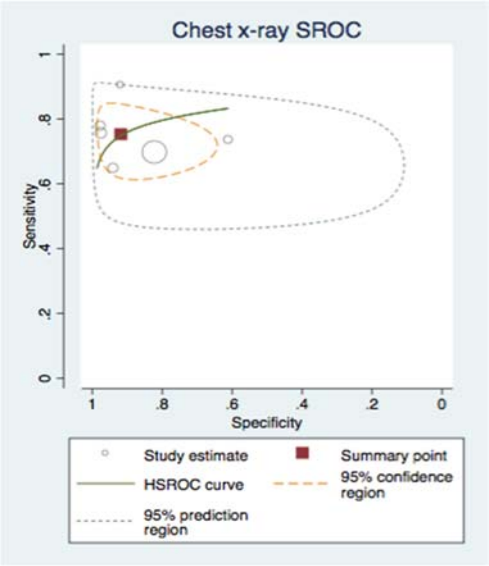

©2019 Maw AM et al. *JAMA Network Open.*
